# Diversity and effect of *Trichoderma* isolated from the roots of *Pinus densiflora* within the fairy ring of pine mushroom (*Tricholoma matsutake*)

**DOI:** 10.1371/journal.pone.0205900

**Published:** 2018-11-07

**Authors:** Seung-Yoon Oh, Myung Soo Park, Hae Jin Cho, Young Woon Lim

**Affiliations:** School of Biological Sciences and Institute of Microbiology, Seoul National University, Seoul, Republic of Korea; Soonchunhyang University, REPUBLIC OF KOREA

## Abstract

Pine mushroom (PM, *Tricholoma matsutake*) is an important ectomycorrhizal fungus in Asia primarily due to its value as a food delicacy. Recent studies have shown that fairy rings of PM have distinctive fungal communities, which suggests that other fungi influence the growth of PM. *Trichoderma* is a well-known saprotrophic fungus commonly found in pine roots within PM fairy rings; however, little is known about the diversity of *Trichoderma* associated with PM and how these species influence PM growth. This study focused on diversity of *Trichoderma* isolated from pine roots within PM fairy rings and how these species affect the growth of PM isolate. Based on *tef1a* phylogenetic analyses, nine *Trichoderma* species (261 isolates) were identified. *Trichoderma songyi* and *T*. *spirale* were the dominant species, and *Trichoderma* community varied geographically. Growth experiments indicated that metabolites from five *Trichoderma* species had a significant influence on the growth of PM isolates. Metabolites of two *Trichoderma* species increased PM growth, while those of three *Trichoderma* species suppressed the growth. Within the fairy rings, *Trichoderma* that had a positive or neutral effect comprised the majority of *Trichoderma* communities. The results of this study suggest that various *Trichoderma* species co-exist within PM fairy rings and that these species influence PM growth.

## Introduction

Ectomycorrhizal fungi have symbiotic relationships with host plants by supplying nutrients (e.g. nitrogen and phosphorus) and receiving carbohydrates in return [1]. Because the root is also inhabited by other microorganisms, dynamic microbial interactions can occur between ectomycorrhizal fungi and soil microorganisms in the root environment [2, 3]. Recent studies that focused on bacteria-ectomycorrhizal fungi interactions revealed novel features of these inter-kingdom interactions [4, 5]. For example, some bacteria negatively influence ectomycorrhizal fungi, while other bacteria promote growth. However, within the fungi, most studies have focused on the relationship between ectomycorrhizal fungi [6]. Saprotrophic fungi, however, can have a positive or negative impact on ectomycorrhizal fungi [6–9], thus, further investigation into the specific interactions between saprotrophic fungi and ectomycorrhizal fungi will improve our understanding of the general ecology of ectomycorrhizal fungi.

Pine mushroom (PM), *Tricholoma matsutake*, is a valuable ectomycorrhizal fungus associated with Pinaceae and Fagaceae [10, 11]. PM fruiting bodies are highly prized mushroom because of its pine-like aroma that is not obtainable artificially [12]. In autumn, fruiting bodies form on the fairy ring (shiro) where PM hyphae are dominant. Because fruiting bodies only form in natural conditions, a better understanding of the environment near the fairy ring is crucial to understanding the ecology of PM. Previous studies have shown that the biological and physiochemical characteristics are different between the PM fairy ring and adjacent soil [13–15] and between the positions within PM fairy rings likely due to effects of PM hyphae [16–19]. As a biotic environment, co-existing microbial communities may influence PM growth in different ways. Using culture-dependent and -independent methods, previous studies showed that the communities of saprotrophic fungi were different in soil within PM fairy rings compared to adjacent soil [13, 20, 21]. This suggests that PM may have an intimate relationship with saprotrophic fungi in the fairy ring. Diverse saprotrophic fungi were frequently detected in soil within PM fairy ring, and were also detected in the pine roots colonized with PM. You et al. [22] described various saprotrophic fungi in the rootlets of *Pinus densiflora* colonized by PM, with *Aspergillus*, *Chaunopycnis*, *Mortierella*, *Penicillium*, *Phialocephala*, *Talaromyces*, and *Umbelopsis* being commonly detected. Therefore, diverse saprotrophic fungi co-exist with PM not only in the soil, but also in the host root within PM fairy rings.

*Trichoderma* are saprotrophic fungi commonly found in plant-associated environments such as forest soil, roots, and leaves [23]. *Trichoderma* have direct beneficial effects on plants by promoting growth, development, productivity, and resistance to abiotic stress [24]. In addition, *Trichoderma* exhibit indirect effects through suppression of pathogens by secreting antibiotic compounds and mycoparasitic activity on pathogenic fungi [25, 26]. A few earlier studies showed that some *Trichoderma* have a positive influence on ectomycorrhizal fungi [27, 28], while more recent studies revealed that most *Trichoderma* have a negative relationship with ectomycorrhizal fungi [6, 9, 29, 30]. In the case of PM, DGGE profiles of the soil within PM fairy rings showed a positive correlation of presence between *Trichoderma* and PM [13]. Recently, we detected several *Trichoderm*a species from roots within PM fairy rings, and one *Trichoderm*a species commonly isolated was a newly described species, *T*. *songyi* [31]. However, little is known regarding the diversity of *Trichoderma* species associated with PM colonized pine roots and how diverse species influence PM growth.

The main objectives of this study were to investigate the diversity of *Trichoderma* species associated with the roots of *Pinus densiflora* within PM fairy rings and to examine their effect on the growth of PM isolate. *Trichoderma* species were isolated from pine roots within PM fairy rings and identified using the translation elongation factor 1-alpha (*tef1a*) gene which has been shown to have high resolution for *Trichoderma* identification [32]. The effects of *Trichoderma* on PM growth were analyzed by comparing the radial growth of PM isolates in the presence or absence of *Trichoderma* metabolites.

## Materials and methods

### Sampling and isolation

**Fig 1:**
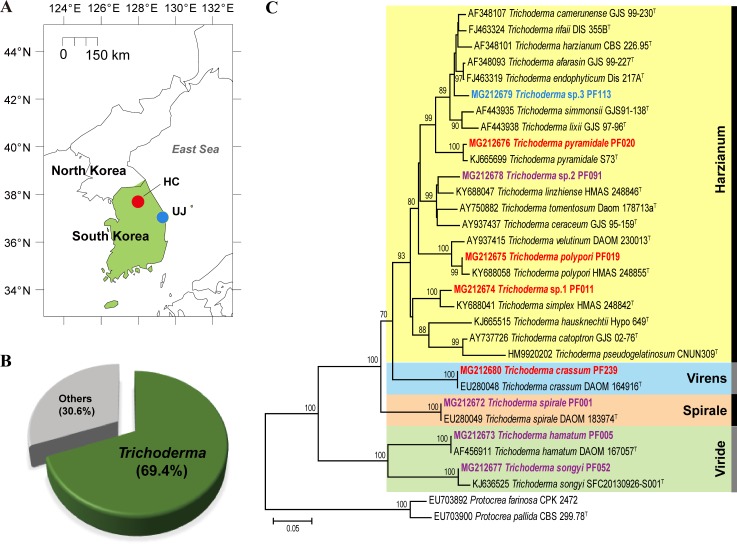
Sampling information and *Trichoderma* diversity isolated from roots of *Pinus densiflora* within PM fairy rings. (A) Map of sampling sites. HC: Hongcheon, UJ: Uljin. Map was made using free vector map data from Natural Earth (http://www.naturalearthdata.com). (B) Relative abundance of *Trichoderma* strains. (C) Phylogenetic tree constructed by neighbor joining method for *tef1a* region from *Trichoderma* species. Bootstrap values of ≥ 70 are represented on the branch. Sequences obtained in this study are represented in bold. Occurrences of *Trichoderma* species in sampling sites are represented by color in species name (red: Hongcheon only, blue: Uljin only, purple: presence in both sites). The letter ^T^ indicates ex-type strain.

Lateral roots of *Pinus densiflora* that were colonized by PM ectomycorrhizae within the fairy ring were collected in September in 2013 from two sites in the Republic of Korea: a research forest that is maintained by the National Institute of Forest Science in Hongcheon County (N37° 41′ 49″ E127° 53′ 19″) and a forested area in Uljin County (N36° 59′ 05″ E129° 06′ 09″) that is known for high PM production (Fig 1A). *Pinus densiflora* was dominant species in both forests, and *Quercus* species were sparsely occurred with *Rhododendron* species as shrub vegetation. Soil type was granite-based sandy soil that was similar to other PM productive sites [19]. All sampling was conducted with permission from the National Institute of Forest Science. The front end of PM fairy ring where the PM hyphae are most actively grown was carefully chosen based on the morphological characteristics and abundance of PM ectomycorrhizae. Six lateral roots (> 10 cm in length) were collected from each of three PM fairy rings at each site. Root samples were transferred to the laboratory in an icebox at 4°C. Soil and organic debris attached to the roots were removed by rinsing with distilled water. Roots were sterilized with 3% NaOCl for 3 min, and washed three times with sterilized distilled water. We confirmed PM colonization on pine roots from ectomycorrhizae attached to lateral root using sequences amplified with PM specific primers [33] and universal fungal primers ITS1F and ITS4 [34]. After detaching ectomycorrhizae, the roots were sliced into pieces of approximately 5 mm in length. Nine root pieces were placed on each Petri dish containing media. Two media were used for isolation: potato dextrose agar (PDA; Difco, USA) was used for isolating fast growing fungi and dichloran rose bengal chloramphenicol agar (DRBC; Difco, USA) was used for slow growing species. Three replicates were used for each fairy ring sample and each media using total of 324 root pieces (9 pieces × 3 plates × 2 media × 3 fairy rings × 2 sites). Plates were incubated at 25°C for 2–7 days. Single fungal strains were generally obtained from each root piece and transferred to PDA medium. If multiple strains, indicated by distinctive morphology, were observed, individual strains were transferred to separate PDA media and subcultured until pure culture was obtained.

### Molecular identification

*Trichoderma* isolates were determined by growth morphologies and microscopic features, and were grouped based on morphological characteristics. Among morphological groups, one to three strains from each *Trichoderma* group were selected as representative if possible. Genomic DNA was extracted from representative strains using a modified CTAB extraction method [35]. PCR was conducted in order to amplify the *tef1a* gene using the primers EF1-728F [36] and TEF1rev [37], using the conditions of Park et al. (2014). PCR products were checked on a 1% agarose gel and purified using Expin^TM^ PCR Purification Kit (GeneAll Biotechnology, Seoul, South Korea). Sequencing was performed at Macrogen (Seoul, South Korea) using an ABI Prism 3730 genetic analyzer (Life Technologies, Gaithersburg, USA).

Sequence analysis for proof reading and editing was conducted using MEGA v. 5 [38]. After aligning sequences using MAFFT v. 7 [39], a phylogenetic tree was constructed using a neighbor joining method and the Kimura-2-parameter model with 1,000 bootstrap replicates. If species identity of representatives were different within morphological groups, we conducted sequence analysis for all strains within the group. Sequences and phylogenetic tree with alignment were deposited at GenBank under accession numbers MG212672-MG212680 and at TreeBASE under accession number S21708, respectively. Community structures were compared by sampling site (Hongcheon vs. Uljin) and culture medium (PDA vs. DRBC) using Constrained Analysis of Principal coordinates (CAP) analysis based on weighted Unifrac dissimilarity with permutational ANOVA test for CAP model.

### Effect of *Trichoderma* metabolites on PM growth

Effects of *Trichoderma* on PM growth were evaluated using a paper disc diffusion method with *Trichoderma* metabolite extract. Metabolites of *Trichoderma* were extracted from each *Trichoderma* species following a previous study [40] with minor modifications of culture conditions and solvent volumes. *Trichoderma* species were cultured on PDA in 90 mm Petri dish for 10 days. Five replicates from each culture plate were chopped in 300 mL of 80% methanol and incubated for a day. After filtering the solution through 150 mm Whatman filter paper (Advantec, Japan), the solvent was concentrated to 10 mL in vacuum using an EYELA rotary vacuum evaporator N-N series (Tokyo Rikakikai, Japan). ‘Tricholoma matsutake’ media (TMM) (glucose 20 g/L, yeast extract 1.5 g/L, soytone 1.5 g/L, and agar 20 g/L) [41] was used for PM growth. We used a single PM strain that was obtained from Korea Mushroom Resource Bank (Seoul, South Korea) (KMRB 12100405). After incubating the PM isolate in potato dextrose broth (PDB; Difco, USA) at 25°C for six months, the PM isolate was homogenized with 30 ml of sterilized distilled water. On the growth media, we inoculated 20 μl of PM isolate at the center of plate. A total of 50 μl of *Trichoderma* extract was inoculated on a sterilized paper disc (8 mm; Advantec, Japan) twice, and air dried in order to evaporate the methanol. Dried paper disc was placed 15 mm away from the center of the plate. All tests were performed in triplicate, and then incubated at 25°C for 1 month. Radial growth (i.e. diameter) of PM isolates was measured twice and averaged. Differences in PM growth was compared between PM cultures with the metabolite disc (treatment) and culture with 80% methanol disc (control). Significance was tested using a pairwise Student t-test adjusted by the false discovery rate of Benjamini and Hochberg [42].

## Results

### Species identification and composition

A total of 376 fungal isolates were obtained from root pieces, and 261 isolates were identified as *Trichoderma* species based on morphological characters (Fig 1B). *Trichoderma* species were isolated from most of the lateral roots within the six fairy rings, and nine distinct species belonging to four clades were identified using *tef1a* sequence analysis (Fig 1C). Six of these were identified at the species level based on the phylogenetic tree, but we were unable to identify three *Trichoderma* species due to ambiguous phylogenetic relationships. The largest number of *Trichoderma* species was found in Harzianum clade (5 species) followed by Viride clade (2 species). *Trichoderma spirale* was the most dominant species (n = 97), followed by *T*. *songyi* (n = 56) and *T*. *hamatum* (n = 52) (Table 1). The number of *Trichoderma* species or strains was similar between culture media. Among non-*Trichoderma* strains, a total of 22 species were identified, and *Penicillium* had the largest number of species (7 species) followed by *Mortierella* (3 species) (S1 Table). The number of strains was largest in *Penicillium* (n = 69) followed by *Umbelopsis* (n = 18) (S1 Table).

**Table 1:** The number of *Trichoderma* strains isolated from roots of *Pinus densiflora* within PM fairy rings.

Species name	Total	Hongcheon			Uljin		
		Total	PDA	DRBC	Total	PDA	DRBC
*Trichoderma crassum*	24	24	13	11	0	0	0
*Trichoderma hamatum*	52	16	7	9	36	18	18
*Trichoderma polypori*	1	1	1	0	0	0	0
*Trichoderma pyramidale*	5	5	2	3	0	0	0
*Trichoderma songyi*	56	8	5	3	48	24	24
*Trichoderma spirale*	97	96	49	47	1	0	1
*Trichoderma* sp. 1	9	9	5	4	0	0	0
*Trichoderma* sp. 2	16	7	0	7	9	3	6
*Trichoderma* sp. 3	1	0	0	0	1	1	0

**Fig 2:**
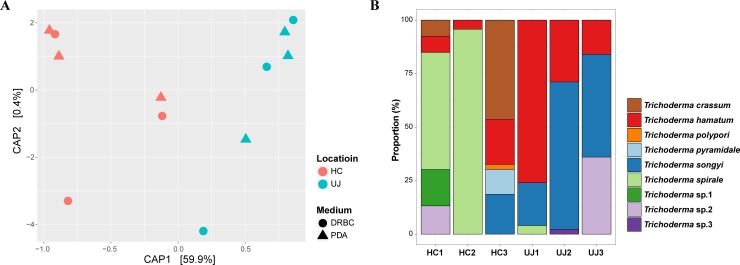
*Trichoderma* community pattern and species composition. (A) Constrained Analysis of Principal coordinates (CAP) plots for *Trichoderma* community structure based on weighted Unifrac dissimilarity. (B) Species composition of *Trichoderma* communities. HC: Hongcheon site, UJ: Uljin site.

CAP analysis based on weighted Unifrac dissimilarities showed that *Trichoderma* communities were significantly different between the sampling sites (*P =* 0.002; 61.5% explanatory power) (Fig 2A), while it were not different between culture media (*P =* 0.961; 0.3% explanatory power). In the Hongcheon samples, *T*. *spirale* (57.8%) was the most dominant species, followed by *T*. *crassum* (14.5%) and *T*. *hamatum* (9.6%) (Fig 2B). In Uljin samples, however, *T*. *songyi* (50.5%) was the most dominant species, followed by *T*. *hamatum* (37.9%). Only four species were isolated from both sites: *T*. *hamatum*, *T*. *songyi*, *T*. *spirale*, and *Trichoderma* sp. 2 (Fig 1C; Table 1). The geographical distribution of non-*Trichoderma* species was different between Hongcheon and Uljin (S1 Table). The number of non-*Trichoderma* strains was higher in Uljin (n = 91) than in Hongcheon (n = 24). At the fairy ring level, the Hongcheon 3 (n = 16) and Uljin 1 fairy rings (n = 37) showed largest number of non-*Trichoderma* strains within each sampling sites. *Umbelopsis nana* was most abundant in Hongcheon (n = 9), and *Penicillium bissettii* was in Uljin (n = 32).

### Effect of *Trichoderma* metabolite on the growth of PM

**Fig 3:**
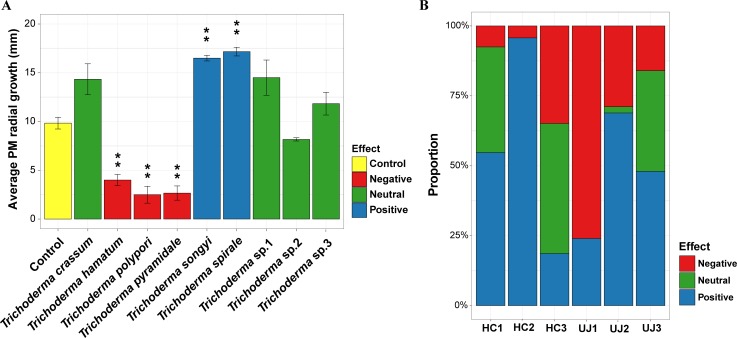
Effect of *Trichoderma* metabolite on PM growth. (A) Average radial growth (mm) of PM grown with metabolite of *Trichoderma* species. (B) Predicted proportion of effect type in *Trichoderma* communities. Growth of PM isolate on treated plates was compared the growth on control plates using pairwise t-tests. An asterisk indicates a significant difference (*P* < 0.01; adjusted by the false discovery rate of Benjamini and Hochberg). HC: Hongcheon site, UJ: Uljin site.

Five *Trichoderma* species showed significant effects on PM growth among the nine *Trichoderma* species (Fig 3A). Two species (*T*. *songyi* and *T*. *spirale*) had a positive effect on PM growth: the treatment increased growth by 168–175% of PM grown alone (control); while three species (*T*. *hamatum*, *T*. *polypori*, and *T*. *pyramidale*) had a negative effect: the treatment decreased growth by 25–41% of control. The *Trichoderma* species showing a positive effect on PM growth were belonging to Spirale (*T*. *spirale*) and Viride clade (*T*. *songyi*) (Fig 1C). The species showing negative effect were belonging to Harzianum (*T*. *polypore* and *T*. *pyramidale*) and Viride clade (*T*. *hamatum*). *Trichoderma* species were categorized based on their effect on PM growth, and the predicted proportion of effect type (i.e. positive or negative effect) in *Trichoderma* communities are presented in Fig 3B. The predicted proportion of *Trichoderma* that had a negative effect was less than 50% in all sampling site, except for the Uljin 1 fairy ring. At the fairy ring level, Hongcheon 3 (34.9%) and Uljin 1 fairy rings (76.0%) had largest proportion of negative *Trichoderma* species within each sampling locations.

## Discussion

### *Trichoderma* diversity in the pine roots under PM fairy ring

*Trichoderma* species are cosmopolitan saprotrophic fungi and are a major component of the mycoflora community in forest soils [43, 44]. Most studies of *Trichoderma* focused on their roles as pathogens [37, 45, 46] or biocontrol agents [23, 47, 48], while relatively little is known about the ecology of individual *Trichoderma* species associated with ectomycorrhizal fungi. Part of the reason for this may be the taxonomic difficulties often encountered within this genus. Traditional taxonomy of *Trichoderma* was based on morphological characters, however, these morphological characters are often insufficient to differentiate species [37] or unreliable because they can change based on environmental conditions [49]. In recent years, sequence analysis of the nuclear ribosomal internal transcribed spacer region (ITS), the second largest unit of the RNA polymerase II (*rpb2*), and the *tef1a* region have improved species identification [50–52] within the *Trichoderma*. Approximately 250 species of *Trichoderma* have been detected based on a combination of genetic and morphological data to date [53].

*Trichoderma* was the most dominant genus in the fungal communities we studied (69.4%) (Fig 1B), which suggests that PM share habitats with *Trichoderma* and they may interact together. A total of nine *Trichoderma* species were identified, and *T*. *hamatum*, *T*. *songyi*, and *T*. *spirale* were isolated most frequently (Table 1). Among these, the initial discovery of the species *T*. *songyi* was in a PM-associated environment: soil and root within the fairy rings and fruiting bodies of PM [31]. Moreover, five *Trichoderma* species (*T*. *polypori*, *T*. *pyramidale* and three unidentified *Trichoderma* species) detected in this study belong to the Harzianum clade exhibiting fungicolous characteristics such as colonizing mushroom-associated environments like fruiting bodies and mushroom composts [54, 55]. In addition, some *Trichoderma* species in the Harzianum clade (e.g. *T*. *harzianum*) showed fungistatic effects on the growth of white button mushrooms (*Agaricus bisporus*) [56]. These negative effects on fungal growth among the several species in the Harzianum clade are consistent with our results of *T*. *polypori* and *T*. *pyramidale* (Fig 3A).

Previous study showed that various saprotrophic fungi co-existed with PM colonized rootlets of *Pinus densiflora*, while *Trichoderma* was not found [22], likely due to difference of sampling strategy; lateral roots were used in this study, while rootlets were used in You et al. [22]. In this study, non-*Trichoderma* species were also isolated (S1 Table) and had similar species composition with previous studies conducting from PM fairy rings [16, 17, 22]. As *Penicillium* was most abundant species in You et al. [22], the number of *Penicillium* species and strains was highest among non-*Trichoderma* species (S1 Table). In addition, *Mortierella* and *Umbelopsis* have been abundantly detected previously from PM fairy ring [16, 20, 22], and we isolated three *Mortierella* and two *Umbelopsis* species. Therefore, the characteristics of fungal species isolated in this study is similar to it of previous studies, except for *Trichoderma* abundance. According to the number of strains (S1 Table), PM fairy rings with low abundance of *Trichoderma* showed high number of non-*Trichoderma* strains, which suggests that antagonistic relationship between *Trichoderma* and non-*Trichoderma* species can be one of the reasons for relatively small number of non-*Trichoderma* species in this study.

*Trichoderma* communities isolated in this study showed geographical differences (Fig 2A). Among the nine species, only four (*T*. *hamatum*, *T*. *songyi*, *T*. *spirale*, and *Trichoderma* sp. 2) were isolated from both locations (Table 1). On the other hand, the distribution of dominant species showed a clear pattern based on geography (Fig 2B). *Trichoderma spirale* was most frequently isolated from the Hongcheon site, while *T*. *hamatum* and *T*. *songyi* were more frequently isolated from the Uljin site. Geographical differences in *Trichoderma* distribution have been detected in previous studies in China and Tunisia, and this spatial variation may be associated with environmental conditions such as climate, soil properties, and vegetation [57, 58]. Soil fungal communities within PM fairy rings were also significantly different depending on geographical location [20], which suggests the importance of geographic effect on the fungal community in the fairy ring of PM.

### Effect of *Trichoderma* metabolite on PM growth

*Trichoderma* have been well studied because of their beneficial interactions with plants [23]. In contrast to their generally positive effects on the plants, most studies suggest that *Trichoderma* usually have a negative effect on other fungi [25, 26, 59]. It has been shown that *Trichoderma* exhibit biocontrol activity in suppressing plant pathogenic fungi (*e*.*g*. *Fusarium oxysporum*, *Pythium ultimum*, and *Rhizoctonia solani*) using cell wall degrading enzymes and secondary metabolites [26]. In addition, *Trichoderma* does seem to have some level of mycoparasitic ability on an arbuscular mycorrhizal fungus (*Glomus intraradices*) via the penetration of mycelium [25]. In the case of macrofungi, *T*. *pleuroti* and *T*. *pleuroticola* cause green mold disease in the oyster mushroom (*Pleurotus ostreatus*) [46]. In this study, three *Trichderma* species, *T*. *hamatum*, *T*. *polypori*, and *T*. *pyramidale*, exhibited strong antifungal effects on PM growth (Fig 3A). Although nothing is known of the antifungal activity and metabolite secretions of *T*. *polypori* and *T*. *pyramidale* because these are recently recorded species [54, 60], *T*. *hamatum* has been applied for the suppression of other fungi [61–63] and also secretes secondary metabolites with antifungal properties (e.g. gliotoxin, isonitrin, and viridiol) [64–66]. Therefore, antifungal substance in secondary metabolites from *T*. *hamatum*, as well as *T*. *polypori* and *T*. *pyramidale*, may suppress PM growth.

Compared to the effects of *Trichoderma* on saprotrophic or pathogenic fungi, the relationship between *Trichoderma* and ectomycorrhizal fungi has been relatively overlooked [6]. Although some *Trichoderma* have positive or neutral relationship with ectomycorrhizal fungi [7, 27, 28], most previous studies suggested that *Trichoderma* suppressed the growth of hyphae and mycorrhization of ectomycorrhizal fungi [6, 9, 29, 30]. Our results showed, however, that the proportion of *Trichoderma* that had a negative effect on PM was low in the fairy rings (Fig 3B). In addition, the PM growth promoting fungi, *T*. *songyi* and *T*. *spirale*, were the dominant species in the Hongcheon and Uljin sites, respectively. Therefore, the relationship between *Trichoderma* and PM is not restricted to negative impacts, as many of these species obviously have significant positive effects. In both sampling locations, PM growth promoting *Trichoderma* species were dominant, except for Uljin 1 which had a large number of *Trichoderma* that inhibit growth. This unusual proportion of effect type in the Uljin 1 fairy ring suggests that the PM in this site may be in poor health or that other fungi act as PM growth promoting fungi. Given that various bacterial genera can promote PM growth [67–69], PM may be benefited by other fungi that are not belonging to *Trichoderma*. However, it needs to pay attention to interpret the proportion of effect type in *Trichoderma* communities because we used single strain from each *Trichoderma* species, thus intraspecific variation of effect on PM growth was ignored in this study. Therefore, a better understanding of the relationship of PM with multiple strains of *Trichoderma* species, as well as non-*Trichoderma* fungi is needed in order to better understanding the overall microbial interactions associated with PM.

### Conclusions

In conclusion, nine *Trichoderma* species were isolated from the roots of *Pinus densiflora* within PM fairy rings. *Trichoderma* was dominant and community structure was significantly influenced by geographical locations, which suggests that PM have an intimate relationship with various *Trichoderma* species. In the PM growth experiment, the metabolites from *T*. *songyi* and *T*. *spirale* had a positive effect on PM mycelial growth. Therefore, our results suggest that saprotrophic fungi can have positive effects on the physiology of ectomycorrhizal fungi, and that introduction of *Trichoderma* metabolites may improve the prospects of successful PM cultivation in the future.

## Supporting information

S1 Table Fungal community composition isolated from roots of *Pinus densiflora* under PM fairy rings. (DOCX)
