# Supplementary material for: Diversity and effect of Trichoderma isolated from the roots of Pinus densiflora within the fairy ring of pine mushroom (Tricholoma matsutake)
Source: PLoS One. 2018 Nov 7;13(11):e0205900. doi: 10.1371/journal.pone.0205900 (PMC6221287; doi:10.1371/journal.pone.0205900)
Supplement: S1 Table — (DOCX) [file pone.0205900.s001.docx]

**S1 Table. Fungal community composition isolated from roots of *Pinus densiflora* under PM fairy rings*.***

| **Taxonomy** | | | | |  | **Hongcheon** | | |  | **Uljin** | | |
| --- | --- | --- | --- | --- | --- | --- | --- | --- | --- | --- | --- | --- |
| **Phy^1)^** | **Class** | **Order** | **Family** | **Species** |  | **HC1** | **HC2** | **HC3** |  | **UJ1** | **UJ2** | **UJ3** |
| A | Dothideomycetes | Botryosphaeriales | Botryosphaeriaceae | *Diplodia alatafructa* |  | 0 | 0 | 0 |  | 0 | 3 | 0 |
|  |  |  |  | *Sphaeropsis sapinea* |  | 0 | 0 | 0 |  | 0 | 1 | 1 |
|  | Eurotiomycetes | Eurotiales | Aspergillaceae | *Aspergillus parvulus* |  | 0 | 0 | 0 |  | 2 | 0 | 0 |
|  |  |  |  | *Penicillium bissettii* |  | 0 | 0 | 2 |  | 15 | 10 | 7 |
|  |  |  |  | *Penicillium daleae* |  | 1 | 1 | 0 |  | 0 | 0 | 0 |
|  |  |  |  | *Penicillium glabrum* |  | 0 | 0 | 0 |  | 5 | 0 | 0 |
|  |  |  |  | *Penicillium nodositatum* |  | 2 | 0 | 0 |  | 4 | 4 | 4 |
|  |  |  |  | *Penicillium ochrochloron* |  | 0 | 0 | 0 |  | 6 | 0 | 6 |
|  |  |  |  | *Penicillium pancosmium* |  | 0 | 0 | 0 |  | 1 | 0 | 0 |
|  |  |  |  | *Penicillium paraherquei* |  | 0 | 0 | 1 |  | 0 | 0 | 0 |
|  | Leotiomycetes | Helotiales | Dermateaceae | *Pezicula radicicola* |  | 0 | 0 | 1 |  | 0 | 0 | 0 |
|  | Pezizomycetes | Pezizales | Pyronemataceae | *Sphaerosporella* sp. |  | 0 | 0 | 1 |  | 0 | 0 | 0 |
|  | Sordariomycetes | Amphisphaeriales | Pestalotiopsidaceae | *Pestalotiopsis rhododendri* |  | 0 | 0 | 2 |  | 0 | 0 | 0 |
|  |  | Hypocreales | Bionectriaceae | *Clonostachys rosea* |  | 0 | 0 | 0 |  | 1 | 0 | 1 |
|  |  |  | Hypocreaceae | *Trichoderma crassum* |  | 4 | 0 | 20 |  | 0 | 0 | 0 |
|  |  |  |  | *Trichoderma hamatum* |  | 4 | 3 | 9 |  | 19 | 13 | 4 |
|  |  |  |  | *Trichoderma pyramidale* |  | 0 | 0 | 5 |  | 0 | 0 | 0 |
|  |  |  |  | *Trichoderma songyi* |  | 0 | 0 | 8 |  | 5 | 31 | 12 |
|  |  |  |  | *Trichoderma spirale* |  | 29 | 67 | 0 |  | 1 | 0 | 0 |
|  |  |  |  | *Trichoderma polypori* |  | 0 | 0 | 1 |  | 0 | 0 | 0 |
|  |  |  |  | *Trichoderma* sp.1 |  | 9 | 0 | 0 |  | 0 | 0 | 0 |
|  |  |  |  | *Trichoderma* sp.2 |  | 7 | 0 | 0 |  | 0 | 0 | 9 |
|  |  |  |  | *Trichoderma* sp.3 |  | 0 | 0 | 0 |  | 0 | 1 | 0 |
|  |  |  | Incertae sedis | *Sarocladium kiliense* |  | 1 | 0 | 1 |  | 1 | 0 | 2 |
| Z | Incertae sedis | Umbelopsidales | Umbelopsidaceae | *Umbelopsis isabellina* |  | 0 | 0 | 0 |  | 0 | 0 | 2 |
|  |  |  |  | *Umbelopsis nana* |  | 3 | 0 | 6 |  | 2 | 1 | 4 |
|  | Mortierellomycetes | Mortierellales | Mortierellaceae | *Mortierella alpina* |  | 0 | 0 | 0 |  | 0 | 0 | 1 |
|  |  |  |  | *Mortierella gamsii* |  | 0 | 0 | 1 |  | 0 | 0 | 0 |
|  |  |  |  | *Mortierella verticillata* |  | 0 | 0 | 0 |  | 0 | 0 | 3 |
|  | Mucoromycetes | Mucorales | Cunninghamellaceae | *Gongronella koreana* |  | 0 | 0 | 1 |  | 0 | 0 | 1 |
|  |  |  | Mucoraceae | *Mucor zonatus* |  | 0 | 0 | 0 |  | 0 | 0 | 3 |

^1)^ Phy: Phylum (A: Ascomycota, Z: Zygomycota)
